# Use of an anionic collagen matrix made from bovine intestinal serosa for *in vivo* repair of cranial defects

**DOI:** 10.1371/journal.pone.0197806

**Published:** 2018-07-12

**Authors:** Mariane Silva Pettian, Ana Maria de Guzzi Plepis, Virginia da Conceição Amaro Martins, Geovane Ribeiro dos Santos, Clovis Antônio Lopes Pinto, Ewerton Alexandre Galdeano, Amanda Regina Alves Calegari, Carlos Alberto de Moraes, Marcelo Rodrigues da Cunha

**Affiliations:** 1 Department of Morphology and Pathology, Faculty of Medicine of Jundiaí, São Paulo, Brazil, Jundiaí –SP, Brazil; 2 Interunit Postgraduate Program in Bioengineering, University of São Paulo, USP, São Carlos-SP, Brazil; 3 Institute of Chemistry of São Carlos, University of São Paulo, USP, São Carlos-SP, Brazil; 4 Department of Anatomical Pathology, A.C. Camargo Cancer Center, São Paulo, SP, Brazil; Università degli Studi della Campania, ITALY

## Abstract

Polymeric biomaterials composed of extracellular matrix components possess osteoconductive capacity that is essential for bone healing. The presence of collagen and the ability to undergo physicochemical modifications render these materials a suitable alternative in bone regenerative therapies. The objective of this study was to evaluate the osteogenic capacity of collagen-based matrices (native and anionic after alkaline hydrolysis) made from bovine intestinal serosa (MBIS). Twenty-five animals underwent surgery to create a cranial defect to be filled with native and anionic collagen matrixes, mmineralized and non mineralized. The animals were killed painlessly 6 weeks after surgery and samples of the wound area were submitted to routine histology and morphometric analysis. In the surgical area there was new bone formation projecting from the margins to the center of the defect. More marked bone neoformation occurred in the anionic matrices groups in such a way that permitted union of the opposite margins of the bone defect. The newly formed bone matrix exhibited good optical density of type I collagen fibers. Immunoexpression of osteocalcin by osteocytes was observed in the newly formed bone. Morphometric analysis showed a greater bone volume in the groups receiving the anionic matrices compared to the native membranes. Mineralization of the biomaterial did not increase its osteoregenerative capacity. In conclusion, the anionic matrix exhibits osteoregenerative capacity and is suitable for bone reconstruction therapies.

## Introduction

Regenerative medicine currently has several treatment techniques to stimulate the formation of a new bone tissue necessary for the adequate repair of a bone lesion, through the use of cellular therapies, morphogenetic signals and biomaterials. The association of these techniques is essential for the success of a bone regeneration [1]. To date, studies have been done on the use of stem cells in bone regeneration [2,3] as well as in the application of biomaterials as new tools for tissue engineering to substitute the traditional therapies based on autologous graft use [4]. Thus, bone tissue engineering strategies using in vitro and in vivo research are essential for the development of regenerative medicine

Biomaterials used in regenerative therapies should enable good adhesion of cells to the material surface [5], should be non-oncogenic, hemostatic, sterilizable and easily produced on a large scale, and their costs should be acceptable [6]. In addition, these materials should stimulate or guide undifferentiated cells to phenotypically convert into osteoprogenitor cells, which are essential for bone neoformation during repair of bone defects [7]. Given these requirements, some biomaterials such as natural collagen polymers have been extensively used in tissue engineering because of their ability to mimic extracellular matrix reactions and to participate in the control of the cellular phenotype [8].

Collagen is the most abundant protein in the animal kingdom and exerts important biological functions, such as tissue formation and cell attachment and proliferation. Collagen scaffolds provide excellent biocompatibility, controllable biodegradability, flexibility, and the capacity to absorb body fluids for nutrient delivery. Therefore, collagen is often chosen as a biomaterial for the repair of skeletal defects [9–12] since collagen type I is the most abundant component of the organic phase of bone [13]. Its biocompatibility, biodegradability, bioabsorbability, low antigenicity and easy manipulation into different forms make collagen an alternative source in medical applications [14]. Collagen-based matrices made from bovine and equine tissue, especially those containing type I collagen, are the most widely used materials in clinical applications because of their biological properties [15,16].

Collagen also plays an important role in the formation of tissues and organs and has been used as a support for proteins that induce bone formation [17]. In addition, collagen influences cell differentiation and possesses recognition sites that permit interactions with cells [18], stimulating cell migration and infiltration [19].

Special attention is given to collagen in the form of artificial extracellular matrices (aECM) that simulate the interstitial environment of live tissues, assisting in the adhesion, localization and release of cells and growth factors to specific sites of the body. Furthermore, these matrices serve as a three-dimensional support for the formation of new tissues and guide the development and differentiation of new cell types [19]. Various collagen-based aECM were developed from bovine pericardium and tendon. However, another promising material for tissue regeneration is collagen derived from bovine or porcine small intestinal submucosa (SIS). This material has been shown to be rapidly absorbed and can be used as a scaffold to support the constructive remodeling of some body tissues, including musculoskeletal structures, skin, abdominal wall, dura mater, bladder, and blood vessels. This biomaterial is a resistant and malleable tissue that can be used to support cell proliferation during repair and to replace damaged tissues [20]. Lin et al. [21] demonstrated the low immunogenicity of aECM derived from porcine SIS and its capacity to increase angiogenesis through the release of vascular endothelial growth factor (VEGF) during *in vivo* biodegradation. In an *in vitro* study, Rong et al. [22] demonstrated that SIS pre-seeded with endothelial progenitor cells can be applied as an alternative scaffold material in artificial blood vessel prosthesis.

Another advantage of collagen-based aECM is the capacity to undergo changes in their physicochemical properties, regardless of their origin. Such changes are obtained by alkaline treatment that induces the selective hydrolysis of asparagine and glutamine carboxamide side chains, increasing the number of negative charges on the collagen molecule and rendering it a polyanionic polymer [23,24]. This process increases the piezoelectric properties of collagen fibers, which is important for osteogenesis and removes bovine or porcine cells that could cause immune rejection [25]. Within this context, the objective of this study was to evaluate bone neoformation during the repair of cranial defects grafted with anionic collagen matrices derived from bovine small intestinal serosa (MBIS).

## Materials and methods

### Preparation of collagen matrices derived from bovine intestinal serosa (MBIS)

The Bovine intestinal serosa, obtained in the butcher's shop, was washed in 0.9% saline (NaCl) and distilled water. The membrane was then divided into two fractions, called native matrix and anionic matrix.

After cleaning, the native matrix was equilibrated in 0.01 mol L^-1^ H_3_PO_4_ for 24 h to permit swelling, washed in deionized water, lyophilized, and stored at 25°C. For preparation of the anionic matrix, the clean serosa was placed in alkaline solution for 24 h at a temperature of 25°C. The hydrolysis solution consisted of salts (sulfate and chloride) and alkali and alkaline earth metal hydroxides, and the routine procedure of the Group of Biochemistry and Biomaterials, Institute of Chemistry of São Carlos, University of São Paulo, was followed [26]. After this period, the serosa was equilibrated in a solution containing Na^+^, K^+^ and Ca^2+^ sulfates and chlorides. Excess salts were removed through washing in a solution of 3% boric acid, 0.3% EDTA and deionized water. After hydrolysis, the anionic matrix was equilibrated in a solution of 0.01 mol L^-1^ H_3_PO_4_ and deionized water for 24 h, lyophilized, and stored at 25°C.

The native and anionic matrices were divided into two new fractions. One fraction was equilibrated in phosphate-buffered saline (PBS, pH 7.4), washed in deionized water, and again lyophilized. The other fraction was mineralized *in vitro*. The following four matrices were thus obtained: 1) non-mineralized native bovine serosa (NBIS); 2) mineralized native bovine serosa (MNBS); 3) non-mineralized anionic bovine serosa (BS24), and 4) mineralized anionic bovine serosa (MBS24).

### Mineralization by alternate immersion

*In vitro* mineralization was performed by alternate immersion in 0.2 mol L^-1^ CaCl_2_ (pH 7.4) and 0.12 mol L^-1^ Na_2_HPO_4_ (pH 9.0) for 30 min each at 37°C, repeating the process six times. After mineralization, the matrices were washed in deionized water and lyophilized.

### Characterization of the matrices

*Differential scanning calorimetry (DSC)*: The analyses were performed with a DSC 2010 calorimeter (TA Instruments). Approximately 20 mg of the sample was placed in a hermetic aluminum sample pan under a nitrogen flow of 80 mL min^-1^, with a heating rate of 10°C min^-1^ and temperature variation of 5 to 120°C. The denaturation temperature of collagen (Td) was calculated from the midpoint of inflection of the DSC curve.

*Thermogravimetric analysis*: Thermogravimetric curves were constructed to determine the thermal stability of the matrices. Samples of approximately 6 mg in an atmosphere of synthetic air were used, with a temperature variation of 25 to 800°C and a heating rate of 10°C min^-1^ in a TGA Q50 thermogravimetric analyzer (TA Instruments).

*Scanning electron microscopy (SEM)*: SEM photomicrographs were obtained with a Zeiss LEO 440 microscope equipped with an Oxford detector (model 7060) using an electron beam of 20 kV, beam current of 2.82 A, and probe current of 200 pA. The samples were sputtered with a 6-nm layer of gold in a BAL-TEC MED 020 Coating System (BAL-TEC, Liechtenstein) and kept in a desiccator until the time of analysis. The sputtering conditions were a pressure chamber of 2.0 x 10^−2^ mbar, current of 60 mA, and deposition rate of 0.60 nm s^-1^.

*Energy-dispersive X-ray spectroscopy (EDX)*: Analysis was carried out in an EDX Link analytical system (Isis System Series 200) equipped with a SiLi Pentafet detector and ultrathin ATW II (Atmosphere Thin Window), at a resolution of 133 eV to 5.9 keV, coupled to a LEO 440 electron microscope (LEO Electron Microscopy Ltd.) equipped with an Oxford detector (Oxford Instruments, Inc.). A cobalt standard was used for calibration, with an electron beam of 20 kV, focal length of 25 mm, dead time of 30%, beam current of 2.82 A, and probe current of 950 pA.

### *In vivo* experimental design

The study was approved by the Ethics Committee on Animal Experimentation of the Faculty of Medicine of Jundiai (FMJ), São Paulo, Brazil (Protocol No. 327/11). Twenty-five Wistar rats (*Rattus norvegicus*) maintained under appropriate conditions at the Animal House of FMJ were divided into the following groups:

group 1 (control): cranial defect without implant;
group 2 (NBS): cranial defect filled with the non-mineralized native bovine serosa matrix;
group 3 (MNBS): cranial defect filled with the mineralized native bovine serosa matrix;
group 4 (BS24): cranial defect filled with the non-mineralized anionic bovine serosa matrix;
group 5 (MBS24): cranial defect filled with the mineralized anionic bovine serosa matrix.

### Surgical procedure and histology

The animals were anesthetized by gluteal intramuscular injection of an anesthetic solution (1:1) of Coopazine (xylazine) and Dopalen (ketamine) at a dose of 0.01 mg/10 g body weight [27]. After skin incision in the skull cap exposing the parietal bone, a 1-mm thick defect measuring 5 mm in diameter was created with a surgical drill coupled to a hand-held motor [28]. After surgery, the periosteum and skin were repositioned and sutured with 5.0 silk suture. The antibiotic Rifamycin Spray was applied in the cutaneous area of the surgery during the postoperative period and the animals received daily the analgesic Paracetamol diluted in the water of the bottles.

The animals were killed painlessly 6 weeks after surgery with an overdose of the anesthetic, followed by the inhalation of carbon dioxide. The skulls were removed and reduced to the wound area. The samples were submitted to routine histological procedures and 5-μm serial sections were cut and stained with picrosirius red or subjected to immunohistochemistry.

### Immunohistochemistry of osteocalcin expression

Immunohistochemistry was performed using the anti-osteocalcin antibody (sc 30044, Santa Cruz) and EnVision+ System-HRP/DAB kit (K4011, Dako). Endogenous peroxidase was blocked with hydrogen peroxide (10 volumes) and nonspecific binding was blocked with 2% PBS/BSA for 1 h each. For antigen retrieval, the sections were immersed in citrate buffer, pH 6.0, in a water bath. After cooling and washing in PBS, the sections were incubated overnight with the primary antibody at 4–8°C. Next, the sections were washed in PBS and incubated with the secondary antibody for 3 h at 4–8°C. The reaction was developed with diaminobenzidine (0.5 mg/mL) and liquid hydrogen peroxide (0.005 mL/100 mL) in PBS for 10 min. The sections were washed in PBS, counterstained with Mayer’s hematoxylin, dehydrated, cleared, and coverslipped with Entellan (Merck) [29,30]. The immunoexpression of osteocalcin, indicated by the staining intensity of the brown chromogenic substrate in the cell membrane, occurs through cellular signal transduction induced by receptor-protein interaction, followed by an intracellular signaling cascade determined by the signal of the intermediate antibody. Undamaged parietal bone and kidney specimens were used as negative and positive control, respectively. According to Delmas et al. [31], despite its origin in the bone matrix, osteocalcin can reach the bloodstream and be hydrolyzed in the liver and kidney by metalloproteases. As a negative control of the immunohistochemical reaction, the primary antibody (anti-osteocalcin) was omitted and the reaction was completed. For the positive control, the reaction was completed.

### Analysis of the relative volume of newly formed bone

Stereology was used for this analysis, which permits to determine three-dimensional parameters in a tissue by counting points on a two-dimensional image. A 100-point quadrilateral grid connected to the microscope was used to calculate the bone volume in the area of the defect. The volume of newly formed bone was quantified according to the principle of Delesse [32], using the formula Vv = Pp/Pt (%), where Vv is the volume density, Pp is the number of points (line intersection) on newly formed bone, and Pt is the total number of points of the system

## Results

### Physicochemical characterization of the matrices

**Fig 1:**
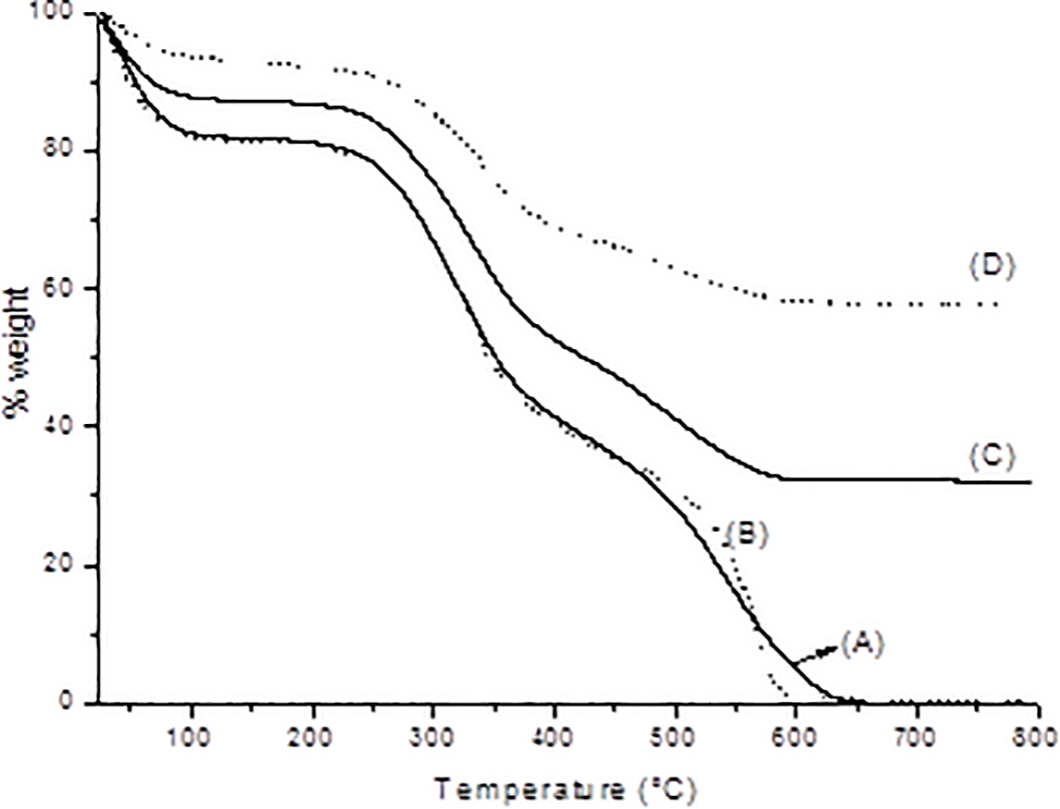
Thermogravimetric curves obtained for the different collagen matrices: (A) NBS, (B) BS24, (C) MNBS, and (D) MBS24.

DSC (Table 1) of the samples showed the thermal transition corresponding to the denaturation temperature (Td) of the bovine serosa matrices and of the matrices submitted to alkaline treatment. The Fig 1 shows the thermogravimetric curves obtained for the untreated matrix and matrix submitted to alkaline treatment (24 h) and the respective mineralized matrices.

**Table 1:** Denaturation temperature (Td, ^o^C) of the different collagen matrices.

Matrix	Td (°C)
**NBS**	**67.0**
**MNBS**	**69.2**
**BS24**	**61.9**
**MBS24**	**64.6**

The degradation temperature of the matrices was calculated considering the onset point of the thermogravimetric curves (Table 2). The degradation temperatures of the mineralized matrices were higher than those of the non-mineralized matrices.

**Table 2:** Degradation temperature of the collagen matrices.

Matrix	T_degradation_ (°C)
**NBS**	**259**
**MNBS**	**264**
**BS24**	**269**
**MBS24**	**277**

To determine the content of inorganic matter, i.e., the amount of calcium phosphate deposited on the matrix during the mineralization process, the residues were obtained at 750°C since hydroxyapatite is stable up to 1200°C [33]. The calcium phosphate content was 32.2% for the untreated matrix and 57.7% for the matrix submitted to alkaline treatment. This difference can be explained by the greater affinity of the alkaline-treated matrix for calcium ions because of the larger number of negatively charged groups in the tropocollagen molecules [34].

**Fig 2:**
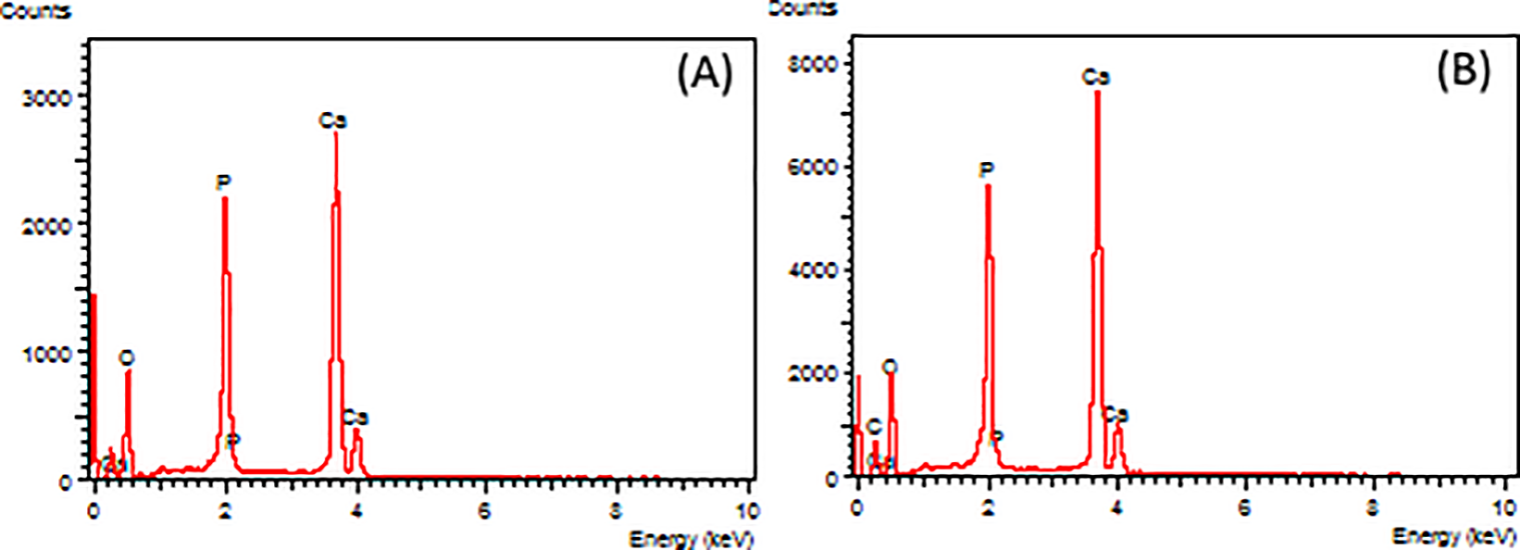
EDX spectra for MBS24 (A) and MNBS (B).

The calcium phosphate formed during mineralization was analyzed by EDX and the calcium to phosphorus (Ca/P) ratio was obtained. The elements found in the matrices were carbon (0.2 keV), oxygen (0.5 keV), calcium (3.7 and 4.0 keV) and phosphorus (2.0 keV), as shown for MBS24 (Fig 2). The Ca/P ratio was 1.64 ± 0.04 for MNBS and 1.80 ± 0.08 for MBS24. The value found for MNBS was similar to that of hydroxyapatite (1.67), while the value obtained for MBS24 indicates a hydroxyapatite rich in Ca.

**Fig 3:**
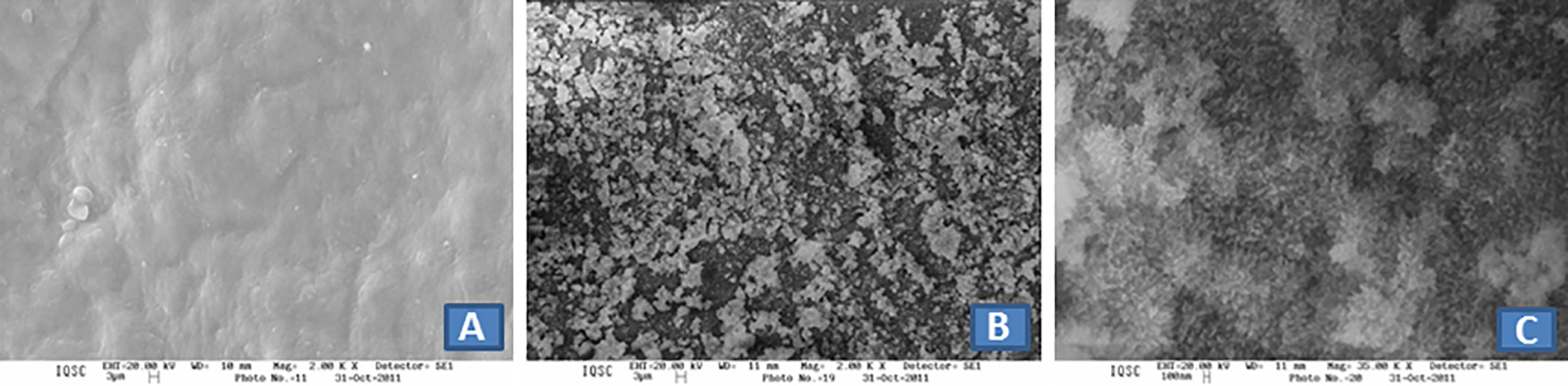
SEM photomicrographs of native bovine serosa: (A) NBS (magnification 2,000x); (B) MNBS (magnification 2,000x); (C) MNBS (magnification 35,000x).

**Fig 4:**
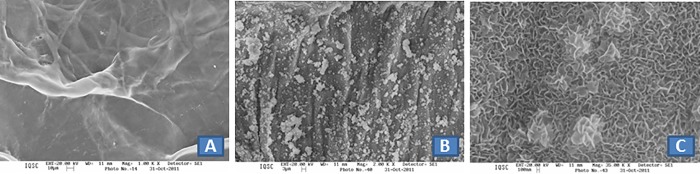
SEM photomicrographs of bovine serosa submitted to alkaline treatment: (A) BS24 (magnification 1,000x); (B) MBS24 (magnification 2,000x); (C) MBS24 (magnification 35,000x).

Analysis of the morphology of non-mineralized bovine serosa revealed alterations in surface rugosity when compared to the mineralized native matrix and the mineralized matrix submitted to alkaline treatment (Figs 3A and 4A). For the mineralized matrix, SEM analysis showed differences in the deposition of calcium phosphate, including the formation of larger aggregates on the native matrix compared to the anionic matrix (Figs 3B, 4B). At 1,000x magnification (Figs 3C and 4C), the morphology of the calcium phosphate crystals is diverse. In the case of the native matrix, the crystals do not have a well-defined shape, while in the anionic matrix they have a needle-like shape similar to that seen in hydroxyapatite.

### Histomorphometric characterization of the bone defect

**Fig 5:**
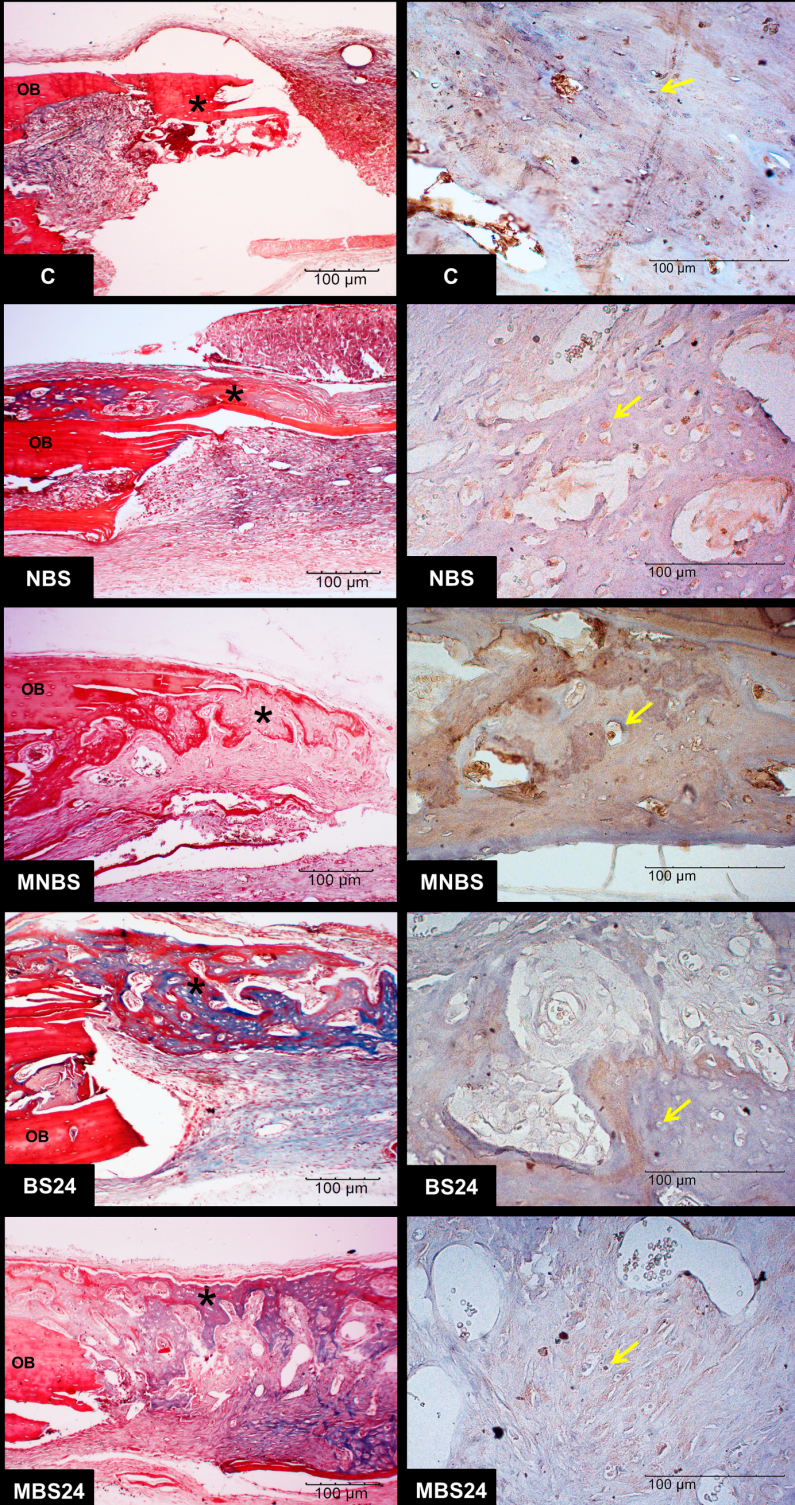
Photomicrographs of the defect area stained with Masson’s trichrome and immunohistochemistry. Note the formation of new bone (*) from the original bone (OB) and osteocalcin staining (brown color) in osteocytes (arrows).

**Fig 6:**
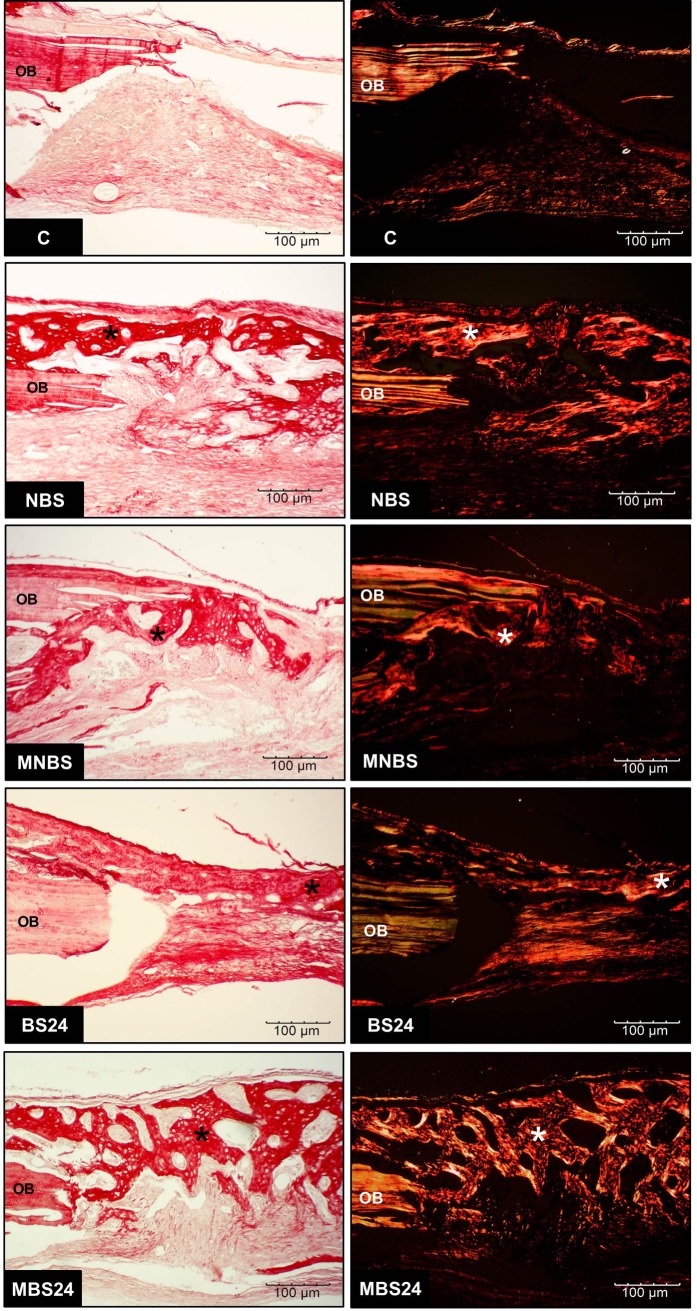
Photomicrographs of the defect area stained with picrosirius red. Observe the birefringence of collagen fibers in the area of newly formed bone (*). OB: original bone.

Formation of new bone projecting from the margins to the center of the defect was observed in all groups. Necrotic tissue was absent due to the presence of bone cells, confirming local neovascularization. No chronic inflammatory infiltrates that would characterize incompatibility of the grafted matrices with the recipient tissue were observed. Bone regeneration was partial as indicated by the presence of areas without bone formation that were invaded by connective tissue. The newly formed bone matrix exhibited trabecular and mature characteristics, harboring osteocytes derived from the differentiation of osteoblasts during formation of the bone matrix. More marked bone neoformation occurred in the BS24 and MBS24 groups in such a way that permitted union of the opposite margins of the bone defect. The presence of various new osteocytes indicated vitality of the newly formed bone. Also in the anionic matrices, there was little connective tissue filling the spaces where new bone had not yet formed. Immunohistochemistry revealed the expression of osteocalcin (characteristic brown staining) in osteocytes lodged inside young bone (Fig 5). The young bone exhibited greater optical density of its collagen fibers (Fig 6).

**Fig 7:**
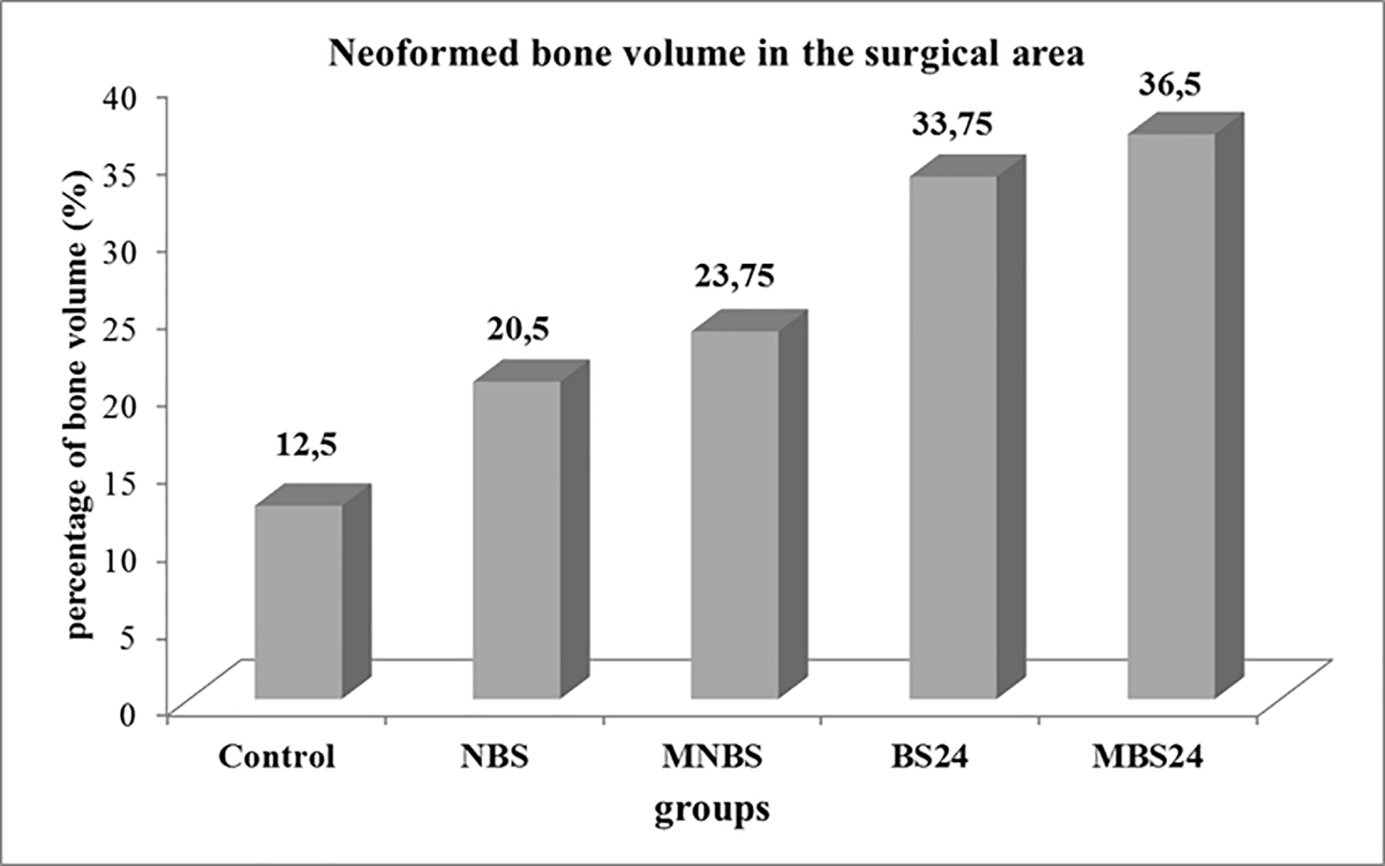
Percentage (%) of newly formed bone in the studied groups.

The mean and standard deviation of the percent volume of bone matrix formed in the cranial defects of the control, NBS, MNBS, BS24 and MBS24 groups sacrificed after 6 weeks were, respectively, 12.5 ± 1.29, 20.5 ± 1.73, 23.75 ± 1.5, 33.75 ± 3.2 and 36.5 ± 1.29. The lowest volume was observed for the control group and there was no significant difference between NBS and MNBS or between BS24 and MBS24. Comparison of the native (NBS and MNBS) and anionic matrices (BS24 and MBS24) showed higher values in the latter (Fig 7).

## Discussion

The repair of bone defects depends on some prerequisites such as the presence of osteoprogenitor cells, vascular and bone growth factors, and support for cell adhesion, growth and proliferation. Synthetic bone substitutes meet these conditions and provide a favorable environment for bone healing similar to that of autologous grafts [35,36]. In this respect, new studies investigating alternative and promising biomaterials for bone regenerative therapies are important.

The cells and extracellular matrix of the small intestine can be used as a xenograft because they provide a microenvironment that is able to stimulate cell differentiation and the release of growth factors without the induction of an immune response [37]. The combination of the extracellular matrix of SIS with other components, such as human mesenchymal stem cells, may be a promising therapeutic resource for cardiac applications [38]. The small intestine is composed of four layers: serosa, muscularis, submucosa (SIS), and mucosa.

The SIS has been studied as a bio-graft for the treatment of different tissues such as the bladder, bowel, heart and bones because of its characteristics such as biocompatibility, rate of controlled degradation, different cell reactions, and matrix-cell interactions [37–43]. On the other hand, little is known about the advantages of matrices derived from the outermost layer of the small intestine, the serosa (MBIS). Therefore, this study evaluated the osteoregenerative capacity of bovine MBIS and determined whether it has a higher affinity for bone tissue receptors than SIS derived from the intestinal submucosal layer. With respect to innovative studies investigating the use of these new synthetic grafts, the evaluation of some characteristics such as biocompatibility and bioactivity is essential for successful experimental and clinical application [44]. According to Anderson et al. [45], inflammation is an inherent response to the mechanism of regeneration when biomaterials are used. In the present study, the collagen matrices derived from bovine intestinal serosa (MBIS) implanted into rat cranial defects were biocompatible, as demonstrated by the histological absence of chronic inflammatory processes that characterize immune rejection of a foreign body. Similar results of biocompatibility have been reported in other studies [40–43,46]. We found no studies in the literature investigating the use of MBIS in bone regeneration.

Cunha et al. [27] and Hirata et al. [47] used a collagen matrix derived from bovine pericardium. The matrix was modified chemically by hydrolysis as done in the present study, which resulted in a negatively charged (anionic) matrix. According to these authors, this treatment transforms the native collagen matrix into a porous structure, which is important for osteogenesis and angiogenesis, in addition to removing cells that could trigger immune rejection when used as an implant. Thus, regardless of their origin, anionic collagen matrices appear to be a good alternative due to their low antigenicity mediated by alkaline treatment.

A reduction in Td values was observed for the alkaline-treated matrices. The same was observed in demineralization studies, i.e., the removal of calcium phosphate from the bone matrix reduces Td, suggesting that the presence of calcium phosphate stabilizes the triple helix [48]. All curves exhibited three events that are related to the loss of water present in the matrix in the range of 25–200°C, decomposition in the range of 200–400°C due to degradation of the structure of the collagen molecule, and carbonization of organic matter in the range of 400–650°C [49].

Hirata et al. [47] also evaluated the capacity of anionic collagen matrices derived from the bovine small intestinal serous layer to stimulate bone repair, similar to the method used in this study. The authors concluded that this biomaterial stimulates osteogenesis, but the bone volume is not sufficient to permit satisfactory repair of the defect. Thus, further research is needed to improve these matrices. In contrast to Hirata et al. [47], we used matrices that were mineralized in order to potentiate osteogenesis, resembling the inorganic matrix of bone tissue.

One of the main challenges in bone tissue engineering is how to provide nutrition and to induce a natural capillary system in the implanted biomaterials [50]. The thin thickness of the SIS permits new cells to survive and to proliferate at the beginning of regeneration through the diffusion of nutrients from the interstitial fluid. Furthermore, the biodegradation of SIS can contribute to the formation of new blood capillaries inside the newly formed bone tissue, suggesting that degradation is important for angiogenesis. This mechanism is related to high concentrations of VEGF in SIS [51]. Thus, SIS used as a scaffold could be a potential biomaterial for bone repair because of its low immunogenicity, biodegradability, and thin and porous structure that is essential for osteogenesis, angiogenesis [21] and osteoinduction [44]. Similar results were observed in this study using MBIS which, unlike SIS, had its structure modified by alkaline treatment.

Modifications in aECM composed of collagen/elastin have been described as an approach to control the *in vivo* degradation of these matrices, providing better integration into the recipient tissue. These alterations include the chemical modification of collagen that results in positively or negatively charged matrices [23,28]. In the present study, macroscopic inspection of the wound area in the groups implanted with the matrices showed fragmentation and absorption of part of the biomaterial, especially of the anionic matrices. The control of resorption is essential for osteogenesis, as indicated by the presence of bone trabeculae projecting from the margins to the center of the defect, permitting a balance between matrix resorption and the formation of new bone. These data agree with König [44] who emphasized the importance of the biomaterial not being absorbed rapidly so that it can concomitantly exert its function as a support and osteoconductor during bone repair.

Another important consequence of the increased negative charges on the anionic collagen matrices are the improved piezoelectric properties of collagen. Groessner-Schreiber et al. [52] showed that positively charged surfaces induced the formation of fibrous connective tissue, while negatively charged surfaces stimulated intramedullary bone formation. Miguel et al. [24] implanted an anionic collagen matrix derived from bovine pericardium into rat calvarial defects and observed biodegradation compatible with bone neoformation and angiogenesis inside the defect. Similar and promising results have also been reported by Cunha et al. [27] who implanted anionic polymer matrices into femoral defects of healthy and ovariectomized rats. Other *in vitro* studies demonstrated that anionic collagen developed as a scaffold to support cultured cells induced the deposition of calcium phosphate salts, production of alkaline phosphatase, and osteoblast differentiation. These results suggest that chemical changes in the collagen matrix may have induced mineralization of the new bone matrix [19].

In the present study, we used native matrices derived from bovine intestinal serosa implanted into animals of the NBS and MNBS groups and matrices treated with alkaline medium for 24 h, corresponding to the anionic matrices implanted into the BS24 and MBS24 groups. The histological results showed more marked bone neoformation at the recipient sites of the anionic matrices compared to the native matrices, corroborating the results of other *in vitro* and *in vivo* studies that used anionic collagen matrices derived from bovine pericardium [19,24,27,53].

Tissue regeneration guided by polymeric collagen matrices is not only the result of the anionic nature of the matrix, but also of the molecular composition of the extracellular matrix of the tissue used for its fabrication and of the combination with other biomaterials [54]. Stereological analysis performed in this study demonstrated the osteoregenerative capacity of anionic matrices derived from bovine MBIS, but no difference in osteogenesis was observed between non-mineralized and mineralized matrices. Ma et al. [55] implanted an SIS matrix combined with different proportions of tricalcium phosphate and hydroxyapatite for the repair of femoral defects in rabbits. The authors concluded that osteogenesis occurs to a greater or lesser extent depending on the quantity of substances used for fabrication of the biomaterial. When compared to the present study using MBIS, it was noted that the extent of mineralization is fundamental for osteogenesis, a fact explaining the negative and unexpected reactions observed for the mineralized matrices in the MNBS and MBS24 groups.

The MBIS used in this study exhibited adequate porosity for osteogenesis and angiogenesis as demonstrated by SEM analysis. However, the impairment of bone neoformation on the mineralized matrices was probably due to excess deposits of calcium phosphate crystals resulting from the mineralization process, which obstructed open pores and the interconnectivity between them. Several studies have shown a direct influence of open microporosity of bone substitutes on the cell response [56,57].

The extracellular matrix of SIS contains proteins that are important for cell differentiation. The collagen content of SIS accounts for more than 90% of the dry weight of the material. The types of collagen present in the intestinal submucosa include types I (predominant), III, IV, and VI. Important glycosaminoglycans are chondroitin sulfate, hyaluronic acid, heparan sulfate, heparin, and fibronectin sulfate [58]. Fibroblast growth factors and VEGF are also found [59]. In view of its composition, the submucosa together with the adjacent connective tissue layer of the small intestine has been shown to be a promising natural biomaterial for tissue engineering, since type I collagen forming a crosslinked fibrillar structure is the most abundant protein of the extracellular matrix and the main organic compound of bone. In addition, collagen ensures the phenotype and organization of cells cultured on this substrate, stimulating the proliferation and differentiation of cells into specific structures of the connective tissue site [42,52].

Some studies have reported the successful use of SIS in regenerative therapies of rat muscle and cartilaginous tissue [60,61]. However, Moore et al. [62] demonstrated unsuccessful bone regeneration when SIS was applied to bone defects in rats. The same observations were made by Zhao et al. [41] who studied pure SIS as a guide for bone repair in rabbits. In contrast, as observed in this study using MBIS, the intestinal serosa layer has adequate capacity to conduct and induce the formation of new bone. Partially agreeing with the studies cited above, Li et al. [63] observed that a biological matrix derived from porcine SIS combined with bone marrow cells induced osteogenesis in dental implants but was not sufficient to promote complete union because of the presence of fibrous tissue at the graft site. The authors concluded that this SIS can act as a scaffold for bone regeneration, but further studies are needed, especially those investigating the culture of osteoblasts on SIS.

Kim et al. [64] implanted a porcine SIS matrix into rat cranial defects and observed significant bone neoformation accompanied by the deposition of collagen and osteocalcin 2 and 4 weeks after implantation. The authors concluded that SIS facilitated bone regeneration. These results are consistent with those of the present study in which birefringence of type I collagen and immunoexpression of osteocalcin were observed in the newly formed bone matrix in areas grafted with the matrices derived from bovine MBIS. In view of the satisfactory bone formation, like SIS, anionic MBIS is also a suitable alternative for bone reconstructive therapies.

## Conclusion

In view of its biocompatibility and osteoconductivity, MBIS can be used for the development of new alternative grafts and may serve as a scaffold in other *in vitro* and *in vivo* studies of reconstructive therapies.
